# Drs. Smith and Tweedie on Fever

**Published:** 1830-04-01

**Authors:** 


### Drs. Smith and Tweedie on Fever.

[Concluded from page 361 of this Number.]

We have dedicated so much space to Dr. Smith's work, in our first article, that we must be brief with him in this. The causes and the treatment of fever remain to be noticed. The immediate or exciting cause, in Dr. Smith's opinion, is a poison formed by the corruption or decomposition of organic matter, vegetable or animal, or both. What this poison is we know not—except that we see it has the power of striking the human being with sickness or death. Of the conditions essential to the putrefactive, or rather febrific, process, heat and moisture are the most certain and perhaps the most powerful. Our author does not appear to be among those who exclude animal matters from the generation of febrific miasms.

"Without doubt, a febrile poison, purely of animal origin, in a high degree of concentration, would kill instantaneously; and when not intense enough to strike with instantaneous death, it would produce a continued fever with the typhoid characters, in the greatest possible degree of completeness and perfection. And this appears to afford the true solution of the origin of the plague. The more closely the localities are examined of every situation in which the plague prevails, the more abundant the sources of putrefying animal matter will appear, and the more manifest it will become, not only that such matter must be present, but that it must abound." 360.

This declaration is followed by an attempt to shew the reason why Grand Cairo is "the birth-place and cradle of the plague." The crowded state of the population—their poverty and nastiness—the narrowness of the streets—the heat of the climate at certain seasons—the filth of the canals, &c. are all arrayed as sufficient causes of the plague. Had Dr. Smith travelled as much as he has read or thought, he would probably have been less positive in his conclusions. The town, or rather the capital of Sion, in the valley of the Rhone, at certain seasons of the year—and so, also, the Jews' quarter on the banks of the Tiber, in Rome, present all the above requisites in a very extraordinary degree—and yet without plague. In the former locality, indeed, we have goitre and cretinism—but, in the latter, we see even less of the common malaria fever than in the fashionable Piazza del Popolo, the Place d'Espagne, or the line of the Pincian and Quirinal hills. It will be evident, however, from the following quotation, that our intelligent author considers the war which has been maintained so long between the *contagionists* and *infectionists* as one merely of words.

"But by far the most potent febrile poison, derived from an animal origin, is that which is formed by exhalations given off from the living bodies of those who are affected with fever, especially when such exhalations are pent up in a close and confined apartment. The room of a fever patient, in a small and heated apartment in London, with no perflation of fresh air, is perfectly analogous to a stagnant pool in Ethiopia, full of the bodies of dead locusts. The poison generated in both cases is the same; the difference is merely in the degree of its potency. Nature, with her burning sun, her stilled and pent-up wind, her stagnant and teeming marsh, manufactures plague on a large and fearful scale; poverty in her hut, covered with her rags, surrounded with her filth, striving with all her might to keep out the pure air and to increase the heat, imitates nature but too successfully; the process and the product are the same, the only difference is in the magnitude of the result. Penury and ignorance can thus at any time, and in any place, create a mortal plague. And of this no one has ever doubted. Of the power of the living body, even when in sound health, much more when in disease, and above all, when that disease is fever, to produce a poison capable of generating fever, no one disputes, and the fact has never been called in question. Thus far the agreement among all medical men, of all sects and of all ages, is perfect.

"But it happens that there is another form of animal matter capable of producing fever: namely, a matter secreted by the living body, constituting not only a poison, but a peculiar and specific poison. This specific poison produces not merely fever, but fever with a specific train of symptoms. In the acknowledgement of this fact, also, the agreement among all medical men is equally perfect.

"But some contend that the poison generated in the first case, and that generated in the second, may both be properly called contagions: others maintain that the application of the same term to two cases so specifically different, destroys a distinction which it is useful to preserve, and that it would be more correct, as well as more conducive to clearness of conception, to call the poison generated in the first case an infection, and to restrict the term contagion, to designate the poison generated in the latter. Vast and immeasurable as the difference appears to be between the contagionists and anti-contagionists, if regard be had merely to their language, yet if attention be paid only to their ideas, to this, and to this only, narrow as the compass is, the whole controversy is reduced. It resolves itself wholly into the question, whether one word shall be used to express two cases which differ from each other in some important circumstances, or whether it may not be more convenient to employ two terms, and strictly to appropriate each to designate its own specific class. It must be manifest that, since both sects are perfectly agreed about the facts, the dispute can be only verbal. If the one would consent to restrict their use of the term contagious, for which there is the best authority and ancient custom, to those diseases which arise from a specific contagion, and would call those which arise from every other poison infectious, there would be an end to this apparently interminable, and in many respects mischievous, controversy." 366.

Of the remote or predisposing causes of fever we need say nothing here—Dr. S. has not attempted to add to our stock of knowledge on this point.

The treatment of fever now only remains to be noticed, and the reader will have pretty well anticipated the principles which Dr. S. is inclined to maintain. The first indication of disorder, he observes, is shewn in the nervous system—it may, he says, possibly be the commencement of inflammation, modified by the nature of the nervous substance, or the peculiar poison exciting the disorder—or it may be "something distinct from inflammation, but having a peculiar tendency to excite it." "In either case (he remarks) the inflammation that is present in fever is peculiar and specific, differing *essentially* from ordinary or simple inflammation." The practical result from this is, that the difference between fever and inflammation is such as requires "a very considerable modification in the treatment appropriate to each." The following proposition is somewhat alarming.

"The only morbid condition of fever, of which we have any knowledge and over which the medical art has any control, is that of inflammation." 376.

Again, he says that although this inflammation be not the primary affection, in the order of events, it is the primary affection in the order of treatment—"if it be not the sole affection that admits of treatment." Still more, he adds, "the remedies proper for febrile inflammation do not differ from those which are adapted to ordinary inflammation—but they differ materially in the mode in which they ought to be applied, and the extent to which they ought to be carried." This, however, is running the parallel between fever and inflammation pretty close—perhaps rather too close for practitioners in general. For if the only difference of treatment consists in certain undefinable modes or proportions of the same remedies in both cases, it is clear that there will be no discrimination at all, in the great majority of cases. We find Dr. Smith opposed to the fashionable doctrine of the surgical part of the profession, who are mostly conversant with symptomatic fever, namely, that a fever may be "put out," as we would put out a fire by a bucket of water, or extinguish a taper by the cone or snuffers. "It is in vain, says he, to hope to terminate fever by a stroke of art." The search after such a remedy is like the pursuit of the philosopher's stone—with this addition, that the physician will often lose his patient during the experiment.

"Fever cannot be cured instantaneously: it may be moderated; it may be gradually subdued; from being violent and dangerous, it may be rendered mild and safe: the physician may bring it to this condition; and this is all that he can accomplish." 377.

Considering the various forms or types of fever, as differing only in degree of intensity, Dr. S. finds it necessary only to state, first of all, the remedies which are appropriate to the disease, and, secondly, the modification of these in the different degrees of intensity. In the first place, he observes, the common continued fevers of this country require little or no treatment. There is no affection of any organ intense enough to need the application of a powerful remedy. The derangement of function is so slight that a spontaneous cure takes place in the course of a few days. Confinement to bed—the abstraction of stimuli—low diet—gentle aperients, repeated according to circumstances, constitute the whole methodus medendi.

But when fever surpasses this mild form it becomes a serious disease. Out of the hundred cases which Dr. S. has detailed, beginning slightly or commencing furiously, take any one or any number, he remarks, and see what is found after death.

"Inflammation, in general, rising in degree, and increasing in extent, or both, in proportion to the intensity of the febrile affection. If this, which may be justly considered as the law of the disease, be not absolutely constant and uniform, it may be safely affirmed, at least, that there are as few apparent exceptions to it, as to any general law that can be named." 379.

The object in practice, then, is clear—to prevent inflammation, or, if that cannot be done, to control or remove it. Accomplish this, he observes, and the fever will not be cured at once—it will go on for some time—but it will come sooner to a close, and proceed mildly to a safe termination. Fail to accomplish this, and the fever will go on from bad to worse—even to the destruction of some organ or organs by the process of inflammation.

Bleeding in fever, he avers, cannot be too soon performed. "The very first moment of excitement, could that be discovered, is precisely the moment when the employment of this powerful remedy would produce the greatest effect. "The earlier the bleeding, the greater will be the impression made upon the disease, and the less upon the patient—the more effectually will the inflammatory action be stopped by the smallest quantity of blood." When inflammation has actually commenced, there is then not a moment to be lost. The inflammation must be stopped—until we do this we do nothing. To effect this reduction of inflammatory action, blood, in greater or lesser quantities, must be drawn. If this golden opportunity be lost, the disease becomes the master of the physician, and, from that moment, he loses control over the malady. A due impression being made on the inflammation by bleeding, the subsequent treatment should consist of purgatives, given to the extent of procuring three or four motions daily—the best purgative being calomel and rhubarb, repeated every night or every second night, followed in the morning by castor oil or the senna draught. Cold sponging of the skin, if hot, acidulated drink, perfect quietude, a dark room, a silent nurse, (if such can be found) and three tea-cupfuls of arrow-root or gruel in the 24 hours—these comprise all else that will be required, or that will be useful, until the period of convalescence.* Such is the simple but most efficient treatment appropriate to the common fever of London and its neighbourhood, (and the author does not speak of fevers which he has not seen) in its ordinary degree of severity. Far as we have carried this analysis, we must make room for one more extract, detailing some particulars of the case of the highly-gifted house-physician of the Fever Hospital—Dr. Dill. We shall make a remark or two after the reader has carefully perused the extract.

"But when the attack commences with severe cerebral affection, the bleeding must be proportionally large, and early as it is copious. A bleeding adequate to subdue a moderate, will be utterly inert in a severe degree of cerebral disease. I give, as a specimen of what may be sometimes required, the case of Dr. DILL. I saw my friend at the very commencement of his attack, and was, therefore, able to carry into effect what I conceive to be the proper treatment with due promptitude and vigour. I saw him *before there was any pain in the head, or even in the back, while he was yet only feeble and chilly.* The aspect of his countenance, the state of his pulse, and the answers he returned to two or three questions, satisfied me of the inordinate, I may say the ferocious, attack that was at hand. *Having taken an emetic without delay, as soon as its operation was over, blood was taken from the arm to the extent of twenty ounces.* During the night severe pain in the limbs, especially in the loins, *and intense pain in the head came on.* The blood that was taken on the preceding evening was not inflamed. Early in the morning he was again bled to the extent of about sixteen ounces, with great diminution, but not entire removal of the pain: the pain not lessening, towards the afternoon he was again bled to the same extent: the pain was now quite gone; the blood from both these bleedings was intensely inflamed.

---

* "It would be trifling, while treating of so momentous a subject as the proper management of fever, which requires the prompt, vigorous, and yet cautious exhibition of the most powerful remedies, to spend any time in discussing the merits of saline, refrigerant, diaphoretic, antimonial medicines, and the rest of the apparatus, which unfortunately continues to hold the place of direct, honourable, and well-earned (if any thing can be well-earned) remuneration to the practitioner." 387.

During the night the pain returned, and, in the morning, the eyes were dull and beginning to be suffused, *while the pulse continued slow and intermittent*, and the respiration suspirious; but the face was blanched, *and the pulse, in addition to its other characters, was weak*. Instead of opening the vein afresh, twelve leeches were applied to the temples; these very much relieved, but still did not entirely remove the pain; for this reason he was cupped to the extent *of sixteen ounces;* this operation afforded very great relief, and he continued easy until the following evening, when the pain returned, and he was again cupped on the temples to the same extent. Immediate relief followed this second operation; *but, unfortunately, the pain returned with great violence towards evening, and it was now impossible to carry the bleeding any farther.* Within twenty-four hours, it was plain that typhoid symptoms in abundance would be present, for the fur on the tongue was becoming brown, and there was already slight tremor in the hands. No more blood could be taken with any prospect of advantage, nor even with safety; yet, without the aid of some powerful remedy the case was lost.

"The whole scalp was now enveloped in ice, but so intense was the heat of the head that it was melted in a few minutes, and the clothes, steeped in the evaporating lotion, dried with extraordinary rapidity. *Neither of these expedients produced the least perceptible effect.*

"What was to be done? Recourse was had to a measure, the efficacy of which is but little known and less appreciated; a remedy the power of which is second only, if, under some circumstances, it be not even superior, to that of the lancet; a remedy which can never supersede the lancet nor dispense with it, but which, when added to it, forms by the combination a treatment so powerful and efficacious that it might render death, from the acutest cerebral inflammation, as rare as recovery is at present.

"This remedy is known by the name of the cold dash. It consists of pouring a column of cold water upon the head in a continued stream from a height of from six to ten feet. The mode of applying it is as follows. The patient is seated in a large tub; a table is placed at the side of the tub upon which a man stands, and at as great an elevation as his arms can reach, pours upon the naked head of the patient a steady but continued stream of cold or iced water, from a watering-pot without the rose. The stream is made to fall as nearly as possible upon one and the same spot. At first the elevation must be slight, for the shock is too violent if the stream be poured at once from the highest point. There is a record that in the East, where ingenuity so long laboured for tyrrany to invent the most exquisite modes of torment, the victim was placed with his bare head under a small stream of cold water, which was so directed as to fall unceasingly upon one spot. In this instance cruelty was cheated of its object by its ignorance of the mode in which its expedient operated. The device was well adapted to kill, but not to produce pain, for insensibility must soon have put an end to suffering.

"Employed as a remedy, there is no degree of burning heat which the animal economy is capable of producing, no intensity of vascular action, and no violence of pain that can resist its continued application. Sooner or later, usually in from ten to twenty minutes, the heat, though most intense, disappears, the skin becomes cold, the face pallid, the features shrunk, while the pulse is reduced to a mere thread, and the pain of the head, however violent and intolerable, entirely ceases. After the patient has been wiped dry, which he should be as rapidly as possible, and placed in bed, the symptoms may soon return in all their violence; the same process will again remove them, and as often as the former recur the latter must be repeated. Three or four repetitions will commonly suffice to subdue the most intense cerebral affection. In the case of Dr. Dill, the relief it brought was instantaneous and most complete. From a state of intense suffering it rendered him perfectly easy, and from a state of imminent danger, safe. I had no anxiety about him from the moment he came out of his tub, although it was necessary to pass him through the same ordeal three times; but he himself having tried this remedy on his sister, having in her case witnessed its efficacy, and now felt it in his own, was extremely desirous that it should be repeated as soon as he was conscious of any return of pain. In consequence of its application, together with the copious depletion that preceded it, at the period when under ordinary treatment, the most exquisite typhoid symptoms would have been present, he was convalescent.* If we consider how powerful the abstraction of caloric must be by every fresh current of water that falls upon the head, to what a mere thread the minute external bloodvessels must be constringed, and consequently to what an extent the internal must be affected, we shall not wonder at its efficacy. Powerful as the cold affusion is when exhibited in its ordinary mode, yet the impression it makes upon the brain, compared with the effect produced by this remedy, may be said to be what the application of six leeches to the temples is to the abstraction of thirty ounces of blood." 403.

Over the mind of the experienced practitioner some doubts, as to the necessity of such vigorous measures, in Dr. Dill's case, will probably have flashed. We have taken the liberty of marking some passages in Italics, in order to draw the reader's attention more specifically to certain points. Knowing the difficulty of conveying, in words, the indications for particular treatment, which are readily recognized by the practised eye of the physician, we should not have noticed this case, but given Dr. Smith full credit for having saved the life of his friend. But having seen Dr. Dill in the midst of this active discipline, when the pulse was slow and intermitting—the respiration suspirious—the face like alabaster—the tongue moist and pale—the skin cold and clammy—we did form an opinion on the case—and that opinion certainly was not in favour of farther depletion. We conceive that Dr. Smith must be endowed with powers of discrimination beyond the usual level of mankind, to foresee the "ferocious attack that was at hand," and that before there was any head-ache, back-ache—nay, when the patient was yet "feeble and chilly." The abstraction of blood in all possible ways, under such circumstances, appears to us now, as it did at the time, a very hazardous experiment, and such as we certainly would not either practise on others, or very cheerfully submit to on ourselves. Dr. Smith may fairly urge his situation of physician to a large fever-hospital, as giving him a preponderating superiority over a physician in private practice—and we grant him all the advantages flowing from such an enviable field for observation. But we may submit that our opportunities of seeing fevers in all their varied forms, in this and in other countries, have not been very limited—nor have we studied their phenomena with a prejudiced eye. We leave the case entirely to the mature reflexions of our brethren, with this one remark, that in this individual instance, Dr. Smith appears to us to have somewhat exceeded the admirable and salutary code of instructions every where conspicuous in the work itself.

---

* "Watchful of the convalescence as experience had taught us it is necessary to be after so severe an attack, still he was allowed to put himself too forward. When to all appearance recovered, though still weak, he undertook a journey of fifty miles, that he might the more completely re-establish his health in the country. He had not arrived at his journey's end an hour before he relapsed. He was again bled, and the cold dash was applied a second time with success. From the commencement to the termination of the disease, 120 pounds (!! surely a mistake) of blood were abstracted in this case."

We have dedicated so much space to Dr. Smith's work that we are unable to notice his chapter on scarlatina, which otherwise might have drawn forth a remark or two. He seems to amalgamate the disease rather too closely with common fever, and thinks its modification arises merely from "its complication with an inflammatory condition of the external and internal skin." He makes no allusion to its cause as a specific contagion, and we apprehend that the treatment recommended will be found rather too active.

Here we must conclude. We shall not wind up with any laboured opinion on Dr. Smith's Treatise, which, like the charge of the judge, on many occasions, only tends to bewilder the jury. But this we will say, that the work just analyzed is the best which we have ever perused, on the subject of fever—and, in our conscience, we believe it the best that ever flowed from the pen of physician in any age or any country. The language is uncommonly elegant—perhaps too metaphorical in some places,—but always perspicuous, and, in the highest degree, illustrative of the ideas and precepts which are meant to be conveyed. The work will establish, on the firmest basis, the talents, the erudition, and the judgment of the author, for ages yet to come.

---

We must now direct some attention to Dr. Tweedie's volume of Clinical Illustrations, in which he has confined himself as much as possible to a detail of facts, and rarely indulges in theoretical discussions. The work is divided into nine short chapters, the first being merely some preliminary observations. In these observations we are glad to see a striking coincidence of opinion respecting the nature of fever, between the two officers of the same institution.

"In every case of genuine fever, there is not one, but several, organs affected; the affection, in the first instance at least, is functional, however soon this functional disturbance may pass into vascular excitement, and afterwards into inflammation.

"Every one who has attentively studied the order of invasion of the symptoms, and more particularly those who have had personal experience of fever, must be satisfied that the brain and nervous system are early and primarily engaged in the febrile action; the disturbance in the brain is, in the beginning, simply functional, though it may, sooner or later, according to particular circumstances, assume an inflammatory character.

"The circulation next partakes in the disorder; there is generally, though not invariably, quick pulse and heat of skin, to which, as a consequence of the previous condition of the sensorium, succeeds a vitiated state of the secretions. Hence the furred tongue, thirst, depraved taste, and turbid urine, observed in fever.

"It is evident, that in this state of febrile excitement, to which the term simple fever may with strict propriety be applied, there is no preponderance of action in any organ; all parts of the system partake equally in the general disturbance. This may continue for an uncertain period, probably for a few days, when it is either brought to a termination by proper measures, or subsides spontaneously.

"When once the torch is lighted, when the circulation is quickened, and the blood consequently impelled with greater velocity through organs whose functions are already disordered, the transition from excitement to inflammation is often rapid. When there is a predisposition to disease in any part, the febrile action is most likely to prey on the organ so predisposed, and the period of the fever at which the inflammation comes on, as well as its intensity, will depend on a variety of concurrent circumstances in each individual case.

"In one instance, we shall find the local affection in the brain; in a second, in the organs of respiration; in a third, in some of the abdominal viscera, most frequently in typhus fever, in the mucous membrane of the intestines; and it not unfrequently happens that more than one of those organs is simultaneously inflamed." 7.

Dr. Tweedie remarks, that this supervening inflammation is of a less intense kind than that of the ordinary phlegmasiæ—though he is unable to account for this modification. The following passage is deserving of notice.

"He who treats complicated fever with the same activity as he would treat any of the phlegmasiæ, is utterly ignorant of one of the most important principles on which the treatment should be conducted." And again, he observes:—

"Fever is not inflammation,—it is, therefore, not cured by remedies that effectually remove the latter, though its violence may be mitigated, and its duration shortened, by the judicious, modified application of the same measures." 8.

This, we conceive, is more judicious advice to the multitude than that which Dr. Smith has conveyed through the medium both of precept and example. Dr. T. looks upon fever as "primarily a general disease," which, in the majority of cases, becomes complicated with local inflammation in the progress of the disorder—the danger being in proportion to the severity of the inflammation—the importance of the organ affected—and the nature of the early treatment. Dr. T. does not, therefore, chime in with any of the LOCALISTS who have figured on the stage, of late years, though he grants that their investigations have had a beneficial effect in drawing attention to the local affections in fever.

The second chapter of the work gives some account of the Fever Hospital—the tables of admission—and a short statistical account of the fever in London, during the last ten years. Of this chapter we can take but very brief notice. In the years 1826 and 7, the fever cases were not only more numerous, but the fever itself was unusually severe. Upwards of 700 cases were rejected for want of room, in the year 1826. The class of patients in the Fever Hospital consists chiefly of the servants of subscribers—of the inmates of work-houses—artisans—and labourers of various descriptions.

The third chapter gives first, some tabular views of admission, cure, and death, for one year, ending the 1st September, 1829. It appears that the admissions during that year were 521, of which 445 were cured—3 discharged into the Small-pox Hospital—and 73 died. From the tables furnished in this chapter it seems, that the period of life most liable to fever, is from 15 to 20 years—next, from 20 to 25—the susceptibility to the disease decreasing as life advances.

Dr. Tweedie classes fever in three divisions, the continued—the periodical—and the exanthemata. These are, of course, subdivided into species and varieties—as, for example, the continued fever, is subdivided into simple, complicated, and typhus. He confines his observations to continued fevers, as those are the forms chiefly admitted into the hospital.

Of simple fever he is convinced that he daily sees examples where there is no evidence of local inflammation—where there is no preponderance of action in any particular organ. Yet, considering how disease creeps on without much external warning in the vital organs, he wisely advises us to watch narrowly for a focus of irritation or inflammation.

In complicated fever, he observes, the local disturbance may be in the head, the chest, or the abdomen—such disturbance constituting the chief source of danger. By typhus fever Dr. Tweedie merely wishes to denote the more severe forms, in which, from the commencement, there is more considerable disturbance in the brain and nervous system—great prostration of strength—with affection of the mucous membranes, &c. He agrees with Dr. Smith, and various other authors, in considering that the primary action of the various exciting causes of fever, is on the brain and nervous system—inflammation being a secondary consequence, and not always taking place at all. In 37 cases out of 54 that were examined after death, the brain shewed evident marks of previous inflammation. In 14 of the fatal cases, "there were no traces of any disease in the brain or its membranes." In these there was destructive inflammation of other organs.

"From this account, we have evidence that the brain and nervous system is very generally, if not universally, involved in the febrile action. This condition of the brain, however, is not to be regarded as the primary cause, but one of the secondary effects of fever. The principle I have so often adverted to, it is important to bear in mind—that the brain being so frequently implicated in fever, the most vigilant measures should be at once adopted, on the very first warning of the approach of inflammation, to prevent those changes of structure which so speedily take place, and render the situation of the patient almost hopeless." 29.

The cerebral affection may be so severe as to be the sole cause of death. It is, however, rarely so. In four cases only was the cerebral disorder so violent as to prove the immediate cause of death. It was only, when the cases were admitted in the early stages, that free depletion was resorted to:—when the symptoms had existed so long as to render vigorous measures doubtful, small local bleedings, blisters, and cold lotions, were employed. Such cases required great discrimination, in subduing the low degree of cerebral inflammation, on the one hand, and husbanding the powers of Nature, on the other. Dr. T. particularly invites his brethren to the observation of a phenomenon which he has often remarked—the masking of the symptoms of other inflamed organs by the cerebral affection. Thus, inflammation of the chest may be entirely overlooked, since, in the absence of cough or difficulty of respiration, there is nothing to lead even to the suspicion of mischief in that quarter. Hence the utility of the stethoscope in such obscure and uncertain cases. Dr. T. has been surprised not to meet with more instances of paralysis, considering the frequency of cerebral inflammation. He only saw one case of it—and that in a man who suddenly lost the use of the right arm, after the more urgent symptoms in the brain had disappeared. He gradually recovered. Retention of urine, however, may be considered as a partial paralysis in this disease, and is by no means infrequent.

"*Affections of the organs of respiration in fever.* Inflammatory affections of the lungs constitute a very common, as well as severe form of complicated fever. This appears from the number of cases in which the pulmonary symptoms were observed, and from which the principal source of danger arose.

"In 103 cases, the lungs were more or less severely affected, of whom about one-third died, which shows the extreme severity of this form of complication.

"In the four months of October, November, December, and January, acute affections of the lungs were very prevalent. Of 187 patients admitted in these months, 61 had some form of chest affection, of whom 53 were bled generally; the average quantity of blood taken from each being about 14 ounces.

"In the spring months the symptoms in the chest were less frequent; but it was observed, that in May, which was unusually cold, pulmonary affections again prevailed; of 56 cases admitted in the course of this month, 14 had severe symptoms of inflammation in the chest." 34.

Since Dr. T.'s appointment to the Fever Hospital, he has seen four individuals die of cynanche laryngea—two of them convalescent from scarlatina. From what he has observed in such cases, he is satisfied that, when the larynx is attacked with acute inflammation (which generally terminates rapidly in œdema of the glottis) the only chance of saving the patient's life, is by the operation of tracheotomy. The operation, we have no doubt, would have saved life in the following case.

"A girl, 13 years of age, after a smart attack of scarlet fever, in which the inflammation in the throat had been very severe, was suddenly seized with symptoms of laryngitis; the breathing was very laborious, and attended with stridulous noise, and frequent attempts to cough, which effort produced the most intense distress. Her deglutition was exceedingly painful, so that she dreaded every attempt to swallow even a little food. The uvula and tonsils were red, but not much swollen; the pulse 140; the skin cool. Leeches had been applied to the throat before I visited her, but with very little relief.

"I proposed that an opening should be made into the windpipe, but this was objected to. Blood was therefore directed to be taken from the arm; a blister to be applied to the throat; and doses of calomel to be taken at intervals. She died, however, eight hours after I saw her, having derived only temporary benefit from those measures.

"On examination of the body, the tonsils were swollen, and from the enlarged mucous ducts a small quantity of bloody puriform fluid escaped; the mucous membrane covering the epiglottis, and the upper portion of the membrane of the larynx, were inflamed and œdematous; but the inflammation not having extended below the rima glottidis, this portion of the tube retained its pale healthy appearance; the parotid, sublingual, and submaxillary glands were enlarged.

"This case affords an instructive lesson; the blood-letting, and other measures evidently hastened the death of the child; and from the healthy condition of the organs below the seat of disease, it is probable that the operation would have saved her life." 37.

Our intelligent author makes some very judicious observations on bronchitis, as an accompaniment of fever. Where patients have suffered previously from what is termed Winter-cough, the case is rendered much more formidable. There is imperfect arterialization of the blood, known by blue colour of the lips, dusky leaden hue of the face and upper extremities, while the functions of the brain become more or less embarrassed, according to the severity of the bronchial affection. The patient becomes delirious—then insensible—the pulse soft and feeble—the tongue covered with deep brown or black crust—while the temperature of the surface falls below par.

True pneumonia occurred in a considerable number of cases, and required the free use of the lancet.

"The remedy, however, in which I placed most confidence, in inflammation of the lungs, but more particularly in bronchitis, either as an auxiliary to bleeding, or when this operation was not justifiable, from the length of time the local symptoms had existed, was the tartar emetic, in doses of one or two grains every second, third, or fourth hour, according to circumstances.

"In general it produced severe vomiting at first, the violence of which was very often lessened by the addition of a few drops of laudanum to each draught; but when the *tolerance* was established, it was most satisfactory to witness the gradual decline of the more urgent symptoms in the chest, and the conviction in the mind of the patient, though much suffering had been endured from the vomiting, when the medicine was first administered, that their amendment was to be ascribed to the remedy." 43.

In a number of instances the inflammation of the chest had been entirely overlooked by the attendant practitioners before the patients entered the hospital—while, in others, it assumed a slow insidious form, without any well-marked symptoms, except a little acceleration of breathing, and slight increase of the fever. "The application of the stethoscope is, in such cases, the only sure method of detecting the state of the lungs; and, under such circumstances, its utility is unquestionable."

Of 523 cases included in this report, 71 had prominent symptoms of abdominal inflammation. Dr. T. conceives, that the morbid condition of the intestinal mucous membrane is one of the *specific effects* of typhus. Of this more anon.

In a large proportion of cases examined after death, a remarkable softness of the spleen was observed, accompanied by a considerable enlargement of the organ. The function of the spleen, however, being unknown, it is useless to draw any conclusions respecting its disorders in fever.

The fourth chapter of the work is on typhus, simple and complicated.

"I should be inclined to include under this term only, those fevers in which the brain and nervous system are early and severely affected, accompanied with symptoms denoting a morbid condition of the mucous membrane and skin, and a tendency to what is known by the term putrescency. This tendency is indicated by the condition of the blood, especially in the advanced stages; the crassamentum of which, instead of forming a firm coagulum, is loose, small in proportion to the quantity of serum, and so soft, that it breaks readily on attempting to raise it, resembling, in consistence, half-boiled currant jelly. In some instances, I have observed, that when blood has been abstracted late in the disease, it scarcely coagulated at all.*

"Not only is the condition of the blood changed in typhus fever, but as a consequence of this morbid state of the blood, the secretions are more or less vitiated. Hence the clammy, disagreeable condition of the mouth—the depraved taste—the dry sordes on the teeth and lips—the brown or black incrustation of the tongue—the peculiar smell from the body, which is easily recognised by those who have much experience in fever, while the excretions are much more fetid than in any other disease of a febrile nature.

"These morbid changes are evidently produced by the action of the febrific poison on the brain and nervous system, and not by its primary operation on the

---

* "See the Lecture of Dr. Clanny, of Sunderland, containing an account of some interesting experiments on the blood in fever."

fluids; in this view alone are the principles of the humoral pathology at all applicable to the phenomena of fever in general." 51.

Simple typhus is considered by our author, as corresponding with the adynamic fever of Pinel and others, being a disorder of function only. It is not often met with in this country; and here Dr. T. enters his protest against the conclusion to which Dr. Burne has come, that this simple typhus is the general character of the continued fever of London. Dr. Burne's description, he avers, only applies to one form of fever in this metropolis.

Dr. T. next adverts to typhus, as complicated with affections of the brain, lungs, and intestines. The latter organs are, he thinks, much more frequently affected in France than in England—though by no means rarely so, even here. Thus, in 54 cases of this fever, there were eight in which inflammation of the mucous membrane was present—sixteen with inflammation and ulceration—in all, 24 out of 54. It is unfortunate, he observes, that we have no diagnostic signs to indicate the existence of gastro-enteritis in fever. We shall often be deceived if we trust to the colour or condition of the tongue.

"I have occasionally been surprised at the precision with which some physicians have spoken of its existence from the red colour of the tongue alone. That this condition of the tongue has been often remarked in cases in which this membrane has been found inflamed after death, accords with my own experience, as well as that of the best informed writers; but, on the other hand, I must state, that I have met with this vermilion colour when no such morbid appearance was discovered after death. In fact, I am disposed to believe that, in many instances, the injection of the tongue is entirely a local affection, and not at all a certain index of the existence of gastro-enteritis." 59.

The general absence of pain is another circumstance deserving of notice. It is not, indeed, to be expected, that the mucous membrane will exhibit much pain, in slow inflammation, when pressure is so difficult of application. It is curious that diarrhœa is far from being a constant attendant of this muco-enteritis, though it might naturally be expected. When the mucous membrane of the colon, however, is inflamed, the symptoms assume the dysenteric character—the discharges being frequent, small, and bloody, accompanied with pain. After death, indeed, the signs of gastro-enteritis are unequivocal enough. The usual phenomena of inflammation, and even of ulceration, are sufficiently apparent. On this important sequela or accompaniment of fever, Dr. Tweedie has favoured his brethren with very many interesting remarks and observations. We must pass over Dr. T.'s notices of petechiæ, erysipelas, gangrene, in order to dedicate more space to the fifth chapter, on the CAUSES OF FEVER.

It was not difficult, our author states, to trace the cause of fever, in many patients who entered the hospital, to cold, intemperance, fatigue, &c. but, as a great number came from certain districts of the metropolis, which are seldom exempt from visitations of fever, it is to be presumed that some local cause existed in such situations. Scarcity of food has long been known to predispose strongly to the operation of other febrific causes; and it is not a little curious—

"That though almost every description of mechanics has been, at some period or other, admitted last year into the Fever Hospital, I do not recollect a single instance of a butcher being sent into the establishment. The exemption of this class of people from the plague, when it last visited London, is mentioned by those writers who described this pestilence; and this immunity goes far to prove, that famine is a powerful predisposing cause." 79.

Atmospheric influence is next taken up by Dr. Tweedie, but this occult cause is likely to remain in obscurity for ages yet to come. A local impure atmosphere, however, is more easily understood. Whenever men are crowded, whether in health or disease, a contaminated atmosphere is generated, and fever results from breathing this air.

The hitherto inexplicable terrestrial poison, malaria, next engages Dr. Tweedie's attention, but he cannot be expected to throw any new light on this abstruse subject. He thinks it is to be regretted that this invisible agent should have been too indiscriminately brought forward to explain the origin of fever in this country.

"It has been a most fortunate loop-hole for some who affect to disbelieve the doctrine of contagion, as one of the many sources of fever. I have been told, that the existence of malaria can be pointed out in particular streets, and in particular houses, which from their situation, ventilation, and construction as to drainage, render such a supposed cause of fever extremely improbable. I have known individuals, on whom such an assumed cause of the disease has had such an impression, that they have absolutely thrown up their houses, and retired from London." 85.

Dr. Tweedie believes in contagion—to which belief he is irresistibly led by the evidence of his own senses. Contagion, however, is only one of the causes of fever, and has, till lately, been too exclusively looked to as the principal cause.

"I have no hesitation, after an impartial inquiry into the subject, and ample means of investigation, to affirm my decided conviction, that fever will spread by contagion; but that the probability of its extension depends very much on cleanliness, the proper ventilation of the sick chamber, and the purity of the surrounding atmosphere." 86.

Dr. T. believes that the contagious principle may be so diluted by pure air, as to be entirely innocuous, just as a mineral acid may, by dilution, be deprived of its caustic qualities. The arguments in favour of contagion may be overstrained by looking to the number of people who are successively or simultaneously attacked by fever. Such occurrences may be explained on the principle that all those who have been attacked, were exposed to the same exciting cause. The following observations bear on the question of contagion.

"The London Fever Hospital is placed in an open space, situate in the vicinity of the metropolis, close to the Small-Pox Hospital. Both these establishments stand in the centre of a large field, where the production of malaria is extremely improbable. I can state, from the most authentic sources, that every physician, with one exception, (the late Dr. Bateman) who has been connected with the Fever Hospital, has been attacked with fever during his attendance, and that three out of eight physicians have died.

"The resident medical officers, matrons, porters, laundresses, and domestic servants not connected with the wards, and every female who has ever performed the duties of a nurse, have one and all invariably been the subjects of fever; and to show that the disease may be engendered by fomites in clothing, the laundresses, whose duty it is to wash the patients' clothes, are so invariably and frequently attacked with fever, that few women will undertake this loathsome, and frequently disgusting duty." 38.

We have reason to believe that Dr. Bateman himself suffered from the fever, and recorded the fact in one of the fever articles written by him in Rees's Cyclopœdia. During Dr. Dill's absence last Summer, in consequence of illness, it was necessary to appoint some person to officiate for him. The first who took up the office, took the precaution of sleeping out of the hospital at night—nevertheless he was attacked with the fever. The duty was then undertaken by a medical pupil, who had completed his education, and was in robust health. He was a stanch non-contagionist, and ridiculed the idea of personal danger from residence in the hospital.

"He performed the duty of house-surgeon for ten days only, when symptoms of a severe fever appeared. Unwilling to believe that he had caught the disease, he ascribed his illness to the effects of common cold, till the febrile prostration, and severe determination to the head, obliged him to resign his duties. He was, within 24 hours, seized with most severe symptoms of cerebral fever, which required the abstraction of nearly 100 ounces of blood, before they were subdued. He passed through a most dangerous attack of fever, and remained in the hospital five weeks, before he could with safety be removed; though I fear this almost fatal personal illustration has not convinced him of the contagious nature of fever." 89.

It has been asserted by the non-contagionists that the Fever-Hospital is surrounded by malaria; but unfortunately the Small-pox Hospital, which actually touches the other, has presented no instance of its officers being affected with fever. Cases of fever are very rare among the nurses of general hospitals, where fever, of course, is not concentrated. But it is useless to multiply arguments in favour of the existence of contagion. Those who have made up their minds on the other side of the question will not listen to reason, and those who are unprejudiced need no further proofs.

The 6th chapter is on the mortality of fever. There is great difference in the ratio of mortality, in different years, situations, and epidemics. In the same institution it has sallied from 1 in 4 to 1 in 12, and that under the same physician—the late Dr. Bateman. Of 50 patients received into Guy's Hospital from May 1816 to April 1817, thirteen died—or 1 in 4. But between May 1817 and April 1818, 258 were admitted, of whom only 16 died—or 1 in 15. After this, it is nearly useless to record the comparative mortality in fever, since absolutely nothing can be drawn from such comparison, in respect to treatment. Dr. Tweedie has, however, collected, with great care and diligence, a number of authentic returns from different hospitals, shewing the comparative mortality, and to these we refer the curious reader.

In the 7th chapter Dr. Tweedie has detailed the history and reduced to a tabular form the morbid appearances observed in 54 cases which were examined after death. These histories and tables we must pass over; but they present a very valuable mass of facts. They corroborate, in all essential points, the statements of Dr. Smith. In the great majority of cases, there was disease in the head, chest, and abdomen.

The 8th chapter embraces the treatment.

"The principles upon which the cases which form the subject of the present report have been treated, it is almost unnecessary to observe, are founded on the conviction, that fever is an acute disease, whether in its simple or complicated form, and therefore requiring antiphlogistic treatment, modified according to the individual circumstances of each case." 165.

"In no case was bleeding more decidedly beneficial than in severe head-ach, with flushing, restlessness, acute delirium, and watchfulness; it often proved the best means of tranquillizing the patient, and inducing sleep. When, however, the delirium was of the low, muttering kind, without pain of head, or flushing, showing a low form of neglected inflammation of the brain, the stage for active treatment had passed over, and experience, therefore, of the doubtful advantage, if not positive injury, resulting from abstraction of blood in such cases, led me to trust to small leechings, cold washes, and blisters to the nape.

"The state of oppression and apparent debility induce the practitioner too often, in such cases, to prescribe a tonic plan of treatment; but it should be remembered, that the low, depressed condition of the patient, is the effect of inflammation in the brain, or its membranes, which will be inevitably increased by the administration of cordials." 167.

The length to which these two fever articles have run prevent us from indulging in more than one other extract, which, however, will prove a very important document.—(See Table annexed.)

"By this statement it appears 280 lost blood—146 from the arm, 70 locally, and 64 both generally and locally; the average quantity of blood drawn was about 19 ounces.

"Of the whole number bled (280), there were

| | Cases. | Average quantity of blood. |
|---|---|---|
| Of Simple Fever | 26 | 8 ounces. |
| Affection of the brain | 110 | 20 |
| ——— chest | 81 | 17 |
| ——— abdomen | 22 | 15 |
| ——— head and chest | 20 | 21 |
| ——— head and abdomen | 12 | 16 |
| ——— head, chest, and abdomen | 9 | 24 |
| | 280 | |

"With respect to the period of the fever at which blood-letting was employed, it may be observed, that although it was chiefly adopted in the early stages, still when the symptoms were urgent, it was abstracted even in the more advanced stages; for it frequently happened that in the course of fever, which at the commencement was comparatively mild, visceral inflammation supervened, which could only be arrested by the lancet." 171.

Dr. Tweedie is not an advocate for indiscriminate depletion, whether by the lancet or other means. He lays down admirable rules, as well as cautions and restrictions in the practical application of the various remedial agents. In short, the present work, concise and unostentatious as it is, would have led us to think that Dr. Tweedie was a man of clear judgment, unfettered by attachment to any fashionable hypothesis—that he was an energetic, but judicious practitioner—and that, if he did not dazzle his readers with the brilliancy of theoretical speculations, he would command their assent to the solidity of his didactic precepts. Such, we say, would be the deductions from the perusal of the work, uninfluenced, we may safely assert, by a personal knowledge that the character of the AUTHOR (which is often otherwise) is not depreciated by intimacy of acquaintance with the MAN.

P. S. We regret that we were unable to allot any space for Dr. Tweedie's section on Scarlatina, which contains much useful and practical advice in respect to treatment.

| 1828 and 1829. | Monthly Admissions. | Numbers bled from the arm. | Number of ounces of blood from the arm. | Number bled locally by cupping or leeches. | No. of ounces of blood locally abstracted, allowing half an ounce taken by each leech. | Numbers bled both generally and locally. | Ounces of blood abstracted: Generally. Locally. | | Monthly Mortality. | Of whom were bled, Generally, Locally, Generally and Locally. |
|---|---|---|---|---|---|---|---|---|---|---|
| September . . . | 60 | 29 | 504 | 8 | 90 | 4 | System. . 132<br>Locally. . 40 | 172 | 12 | Generally . . . . 3<br>Locally . . . . . 2<br>Both . . . . . . . 2<br>—<br>7 |
| October . . . . . | 44 | 13 | 292 | 6 | 61 | 7 | System. . 146<br>Locally. . 89 | 235 | 6 | Generally . . . . 2<br>Locally . . . . . 1<br>Both . . . . . . . 2<br>—<br>5 |
| November . . . | 24 | 5 | 112 | 6 | 37 | 4 | System. . 86<br>Locally. . 33 | 119 | 5 | Generally . . . . 1<br>Locally . . . . . 2<br>—<br>3 |
| December. . . . | 52 | 9 | 188 | 8 | 43 | 7 | System. . 162<br>Locally. . 82 | 244 | 2 | Generally . . . . 1<br>Locally . . . . . 1<br>—<br>2 |
| January. . . . . | 67 | 10 | 218 | 9 | 46 | 6 | System. . 94<br>Locally. . 47 | 141 | 8 | Generally . . . . 1<br>Both . . . . . . . 1<br>—<br>2 |
| February . . . . | 49 | 3 | 28 | 4 | 29 | 5 | System. . 132<br>Locally. . 36 | 168 | 6 | None bled. |
| March. . . . . . | 41 | 13 | 252 | 3 | 25 | 2 | System. . 22<br>Locally. . 19 | 41 | 5 | Locally . . . . . 2<br>Both . . . . . . . 1<br>—<br>3 |
| April. . . . . . . | 28 | 3 | 6 | 6 | 28 | 4 | System. . 56<br>Locally. . 25 | 81 | 2 | Locally . . . . . 1 |
| May . . . . . . . | 56 | 24 | 559 | 9 | 74 | 5 | System. . 112<br>Locally. . 78 | 190 | 9 | Generally . . . . 2<br>Locally . . . . . 2<br>—<br>4 |
| June. . . . . . . | 41 | 18 | 316 | 2 | 16 | 8 | System. . 175<br>Locally. . 109 | 284 | 8 | Generally . . . . 3<br>Both . . . . . . . 1<br>—<br>4 |
| July . . . . . . . | 36 | 12 | 231 | 4 | 42 | 8 | System. . 108<br>Locally. . 64 | 172 | 7 | Generally . . . . 1<br>Locally . . . . . 3<br>Both . . . . . . . 1<br>—<br>5 |
| August . . . . . | 23 | 7 | 131 | 5 | 35 | 4 | System. . 58<br>Locally. . 34 | 92 | 3 | Both . . . . . . . 1 |
| | 521 | 146 | 2892 | 70 | 526 | 64 | System. . 1283<br>Locally. . 656<br>—<br>1939 | | 73 | Generally . . . 14<br>Locally . . . . 14<br>Both . . . . . . 9<br>—<br>37 |

