# Erratum

**Published:** 1830-04-01

**Authors:** 


ERRATUM.

We find we have been led into some error, or rather misconception, at page 390 of this Number, respecting Dr. Dill's case. It appears that it was in a subsequent relapse, not described by Dr. Smith, that we saw Dr. Dill, and expressed our opinion that further depletion was unnecessary. The symptoms, however, were precisely those which Dr. Smith has described—namely the feeble and intermittent pulse, cold skin, blanched face, &c. Our objections to the operation of bleeding, therefore, under such circumstances, are not in the slightest degree altered. The error to which we allude was occasioned by the confused and imperfect account of the case which Dr. Smith has given.

